# Synthesis, crystal structure and Hirshfeld surface analysis of 5-methyl-1*H*-pyrazol-3-yl 4-nitro­benzene­sulfonate at 90 K

**DOI:** 10.1107/S205698902401140X

**Published:** 2024-11-28

**Authors:** Vinaya, Syida A. Yakuth, Thaluru M. Mohan Kumar, Besagarahally L. Bhaskar, Thayamma R. Divakara, Hemmige S. Yathirajan, Yeriyur B. Basavaraju, Sean Parkin

**Affiliations:** a Department of Studies in Chemistry University of Mysore, Manasagangotri Mysuru-570 006 India; b Department of Physical Sciences, Amrita School of Engineering, Amrita Vishwa Vidyapeetham, Bengaluru-560 035, India; c Department of Chemistry, T. John Institute of Technology, Bengaluru-560 083, India; d Department of Chemistry University of Kentucky,Lexington KY 40506-0055 USA; Institute of Chemistry, Chinese Academy of Sciences

**Keywords:** synthesis, crystal structure, Hirshfeld surface, hydrogen bonding, π–π stacking, centrosymmetric tetra­mer

## Abstract

This study presents the synthesis, crystal structure, and a Hirshfeld-surface analysis of the bioactive compound 5-methyl-1*H*-pyrazol-3-yl 4-nitro­benzene­sulfonate­(C_10_H_9_N_3_O_5_S), a pyrazole derivative with pharmacological potential. Pyrazoles are known for diverse bioactivities, and recent research emphasizes their role as a ‘privileged structure’ in drug design. Here, the asymmetric unit of the title compound contains two distinct mol­ecules, *A* and *B*, exhibiting differences in conformation resulting from variation in key torsion angles. These distinctions influence the mol­ecular orientation and inter­molecular inter­actions, with strong N—H⋯N and N—H⋯O hydrogen bonds forming a centrosymmetric tetra­mer stabilized by π–π stacking. Hirshfeld surface analysis readily confirms differing inter­molecular contacts for *A* and *B*, primarily involving hydrogen atoms and differences in their close contacts to nitro­gen and oxygen. This study offers further insight into the mol­ecular architecture and potential inter­actions of pyrazole-based drug candidates.

## Abstract 2

The synthesis, crystal structure, and a Hirshfeld surface analysis of 5-methyl-1*H*-pyrazol-3-yl 4-nitro­benzene­sulfonate (C_10_H_9_N_3_O_5_S), a bioactive compound with pharmacological potential are presented.

## 1. Chemical context

Pyrazoles exhibit diverse pharmacological activities, including protein glycation inhibition, anti­bacterial, anti­fungal, anti­cancer, anti­depressant, anti-inflammatory, anti­tubercular, anti­oxidant, and anti­viral effects (Fustero *et al.*, 2011 ▸; Steinbach *et al.*, 2000 ▸; García-Lozano *et al.*, 1997 ▸). Naim *et al.* (2016 ▸) provide an overview of the current status of pyrazoles and their biological activities. Various reviews focus on bioactive pyrazole derivatives (Ansari *et al.*, 2017 ▸), synthetic and biological attributes of pyrazole compounds (Dwivedi *et al.*, 2018 ▸), and the role of the pyrazole moiety in drug development as a ‘privileged structure’ (Faria *et al.*, 2017 ▸; Patil, 2020 ▸; Yet, 2018 ▸). Comprehensive reviews on pyrazole synthesis and pharmacology are available, highlighting recent advances (Karrouchi *et al.*, 2018 ▸; Fustero *et al.*, 2009 ▸; Ebenezer *et al.*, 2022 ▸).

Several crystal structures of pyrazole derivatives have been reported, including 1,3-diphenyl-4,5-di­hydro-1*H*-pyrazol-5-one (Baddeley *et al.*, 2012 ▸), 1-aryl-1*H*-pyrazole-3,4-di­carboxyl­ate derivatives (Asma *et al.*, 2018 ▸), and additional complex pyrazole compounds (Archana *et al.*, 2022 ▸; Priyanka *et al.*, 2022 ▸; Pintro *et al.*, 2022 ▸; Metwally *et al.*, 2021 ▸). Related structures, such as 5-methyl-1-[(4-methyl­phen­yl)sulfon­yl]-1*H*-pyrazol-3-yl 4-methyl­benzene­sulfonate (XEBLOH) and 1-(4-methyl­phen­yl)-3-phenyl-1*H*-pyrazol-5-yl 4-nitro­benzene­sulfonate, have also been described (Murtaza *et al.*, 2012 ▸; Wardell *et al.*, 2012 ▸).

Given the significance of pyrazoles and specifically 5-methyl-1*H*-pyrazol-3-yl 4-nitro­benzene­sulfonate, this paper presents the crystal-structure analysis of the title compound, C_10_H_9_N_3_O_5_S, **I**.
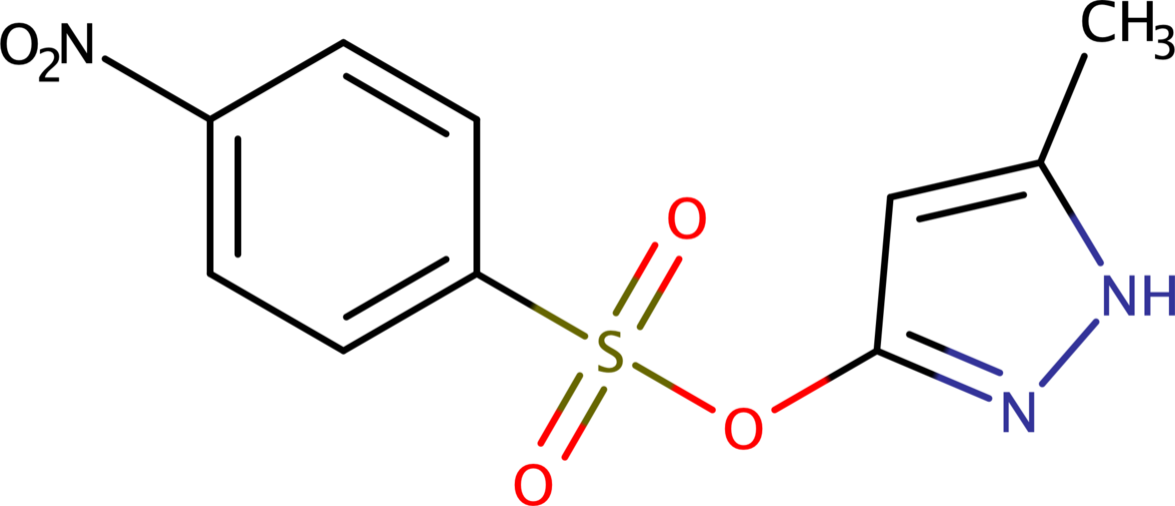


## 2. Structural commentary

**Figure 1:**
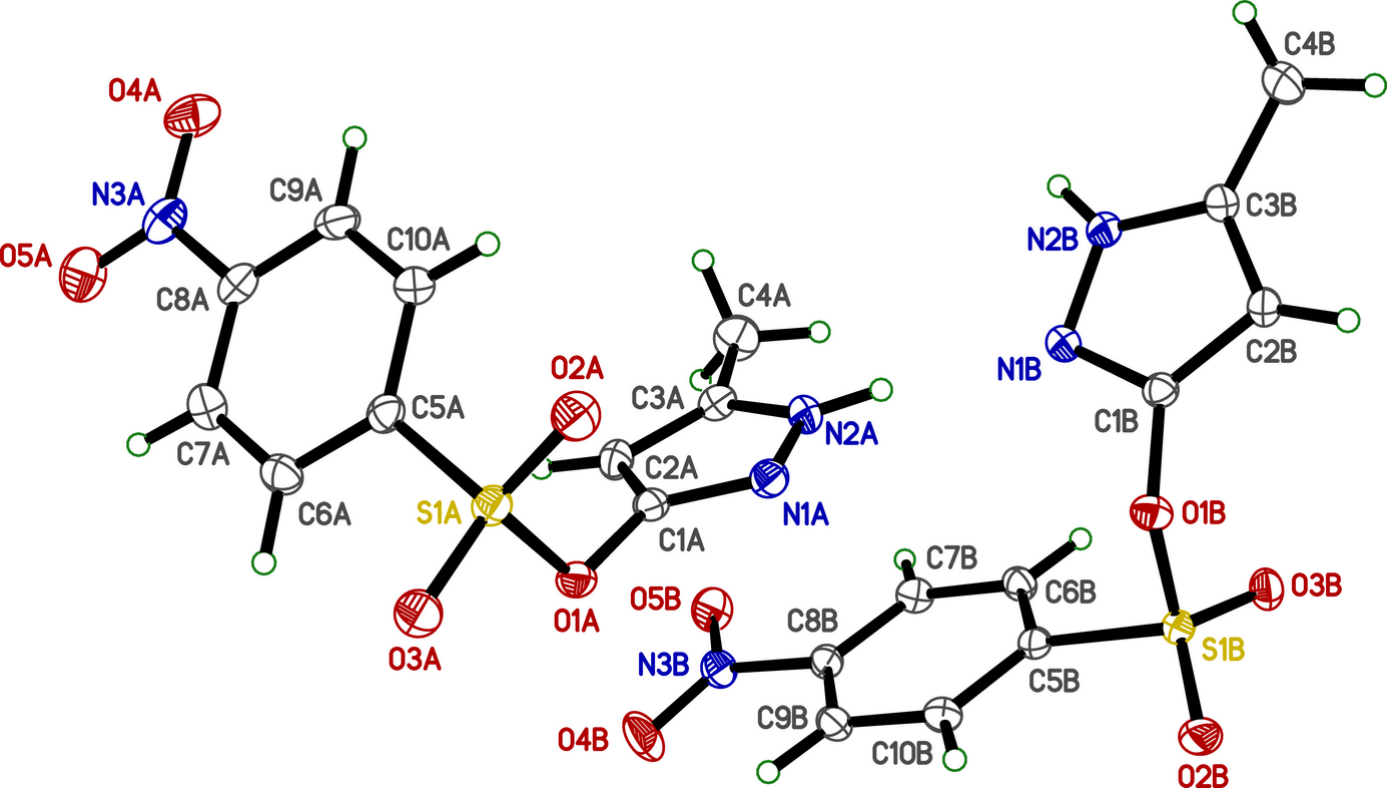
An ellipsoid plot of **I** (50% probability) showing the two crystallographically independent mol­ecules (suffixes *A* and *B*). Hydrogen atoms are shown as arbitrary circles.

**Figure 2:**
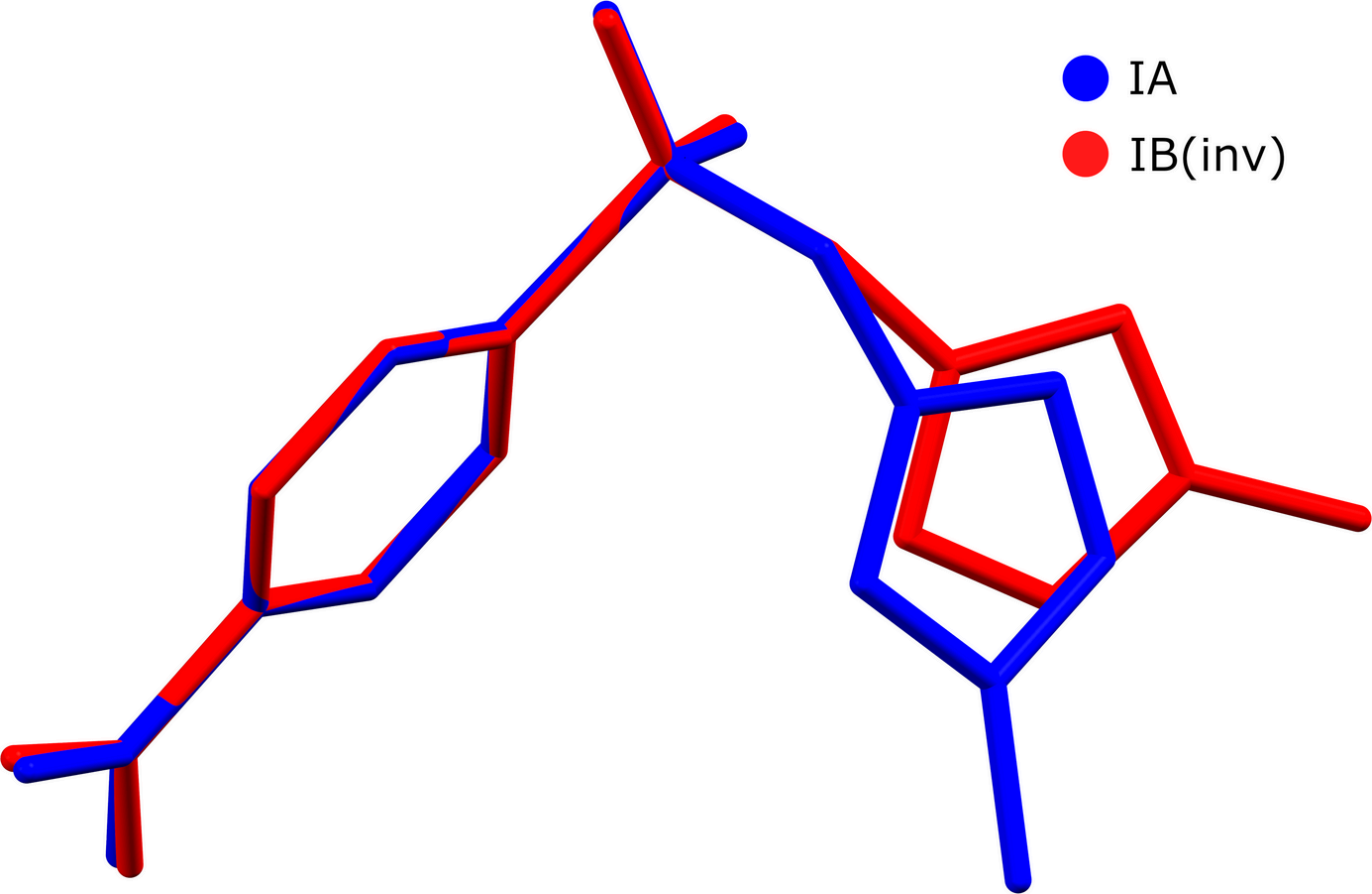
Least-squares overlay of the two mol­ecules of **I**, aligning the benzene rings and the sulfur atom of the sulfonyl group. The coordinates of *B* were inverted for optimal alignment.

The mol­ecular structure of **I** features a 4-nitro­benzene ring bonded to a sulfonate sulfur atom, along with a 3-methyl-1*H*-pyrazole ring attached to the single-bonded oxygen atom of the sulfonate group. The asymmetric unit comprises two crystallographically distinct mol­ecules, *A* and *B* (Fig. 1 ▸). While both mol­ecules exhibit typical bond lengths and angles, their overall conformations differ. The primary distinctions are in the torsion angles N1—C1—O1—S1, C1—O1—S1—C5, and O1—S1—C5—C6, which are 88.97 (12), 64.92 (9), and 78.91 (10)° for mol­ecule *A*, and 83.78 (12), −83.75 (9), and 95.42 (10)° for mol­ecule *B*. These torsional variations lead to differences in the relative proximity and orientation of the pyrazole and benzene rings in each mol­ecule. This is shown in a least-squares fit overlay plot (Fig. 2 ▸) and qu­anti­fied in Table 1 ▸. The only other intra­molecular degree of freedom lies in the rotation of the NO_2_ groups relative to their attached benzene rings. For mol­ecule *A*, this dihedral angle is 1.37 (10)°, *i.e.* nearly coplanar, while in mol­ecule *B*, it is slightly larger at 6.78 (4)°. There are no intra­molecular hydrogen bonds of any type in either mol­ecule *A* or *B*.

## 3. Supra­molecular features

**Figure 3:**
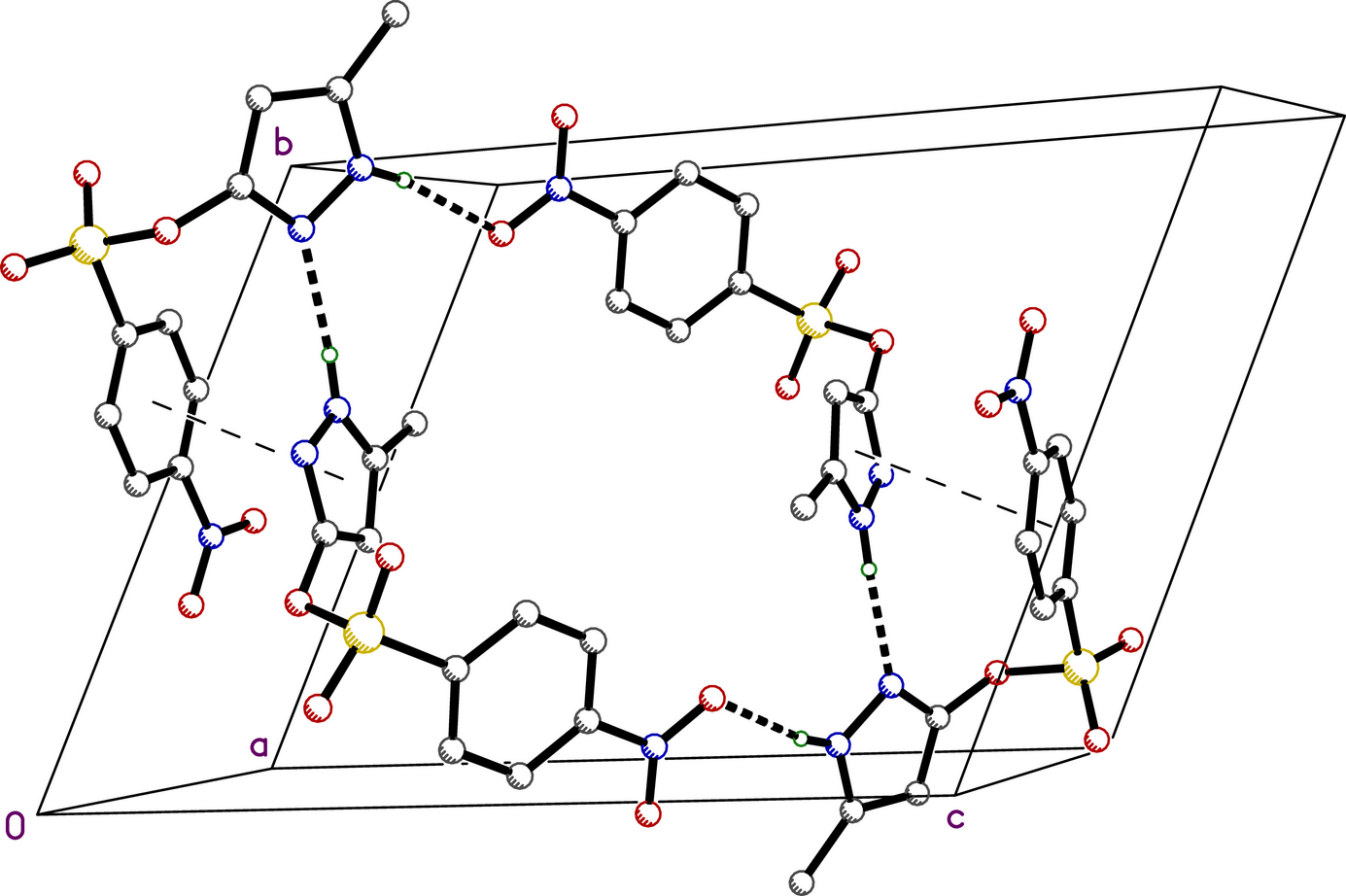
A partial packing plot viewed approximately down the *a* axis. Hydrogen bonds are drawn as thick dashed lines and π–π overlap is shown as thin dashed lines between ring centroids. The whole construct forms a centrosymmetric tetra­mer.

**Figure 4:**
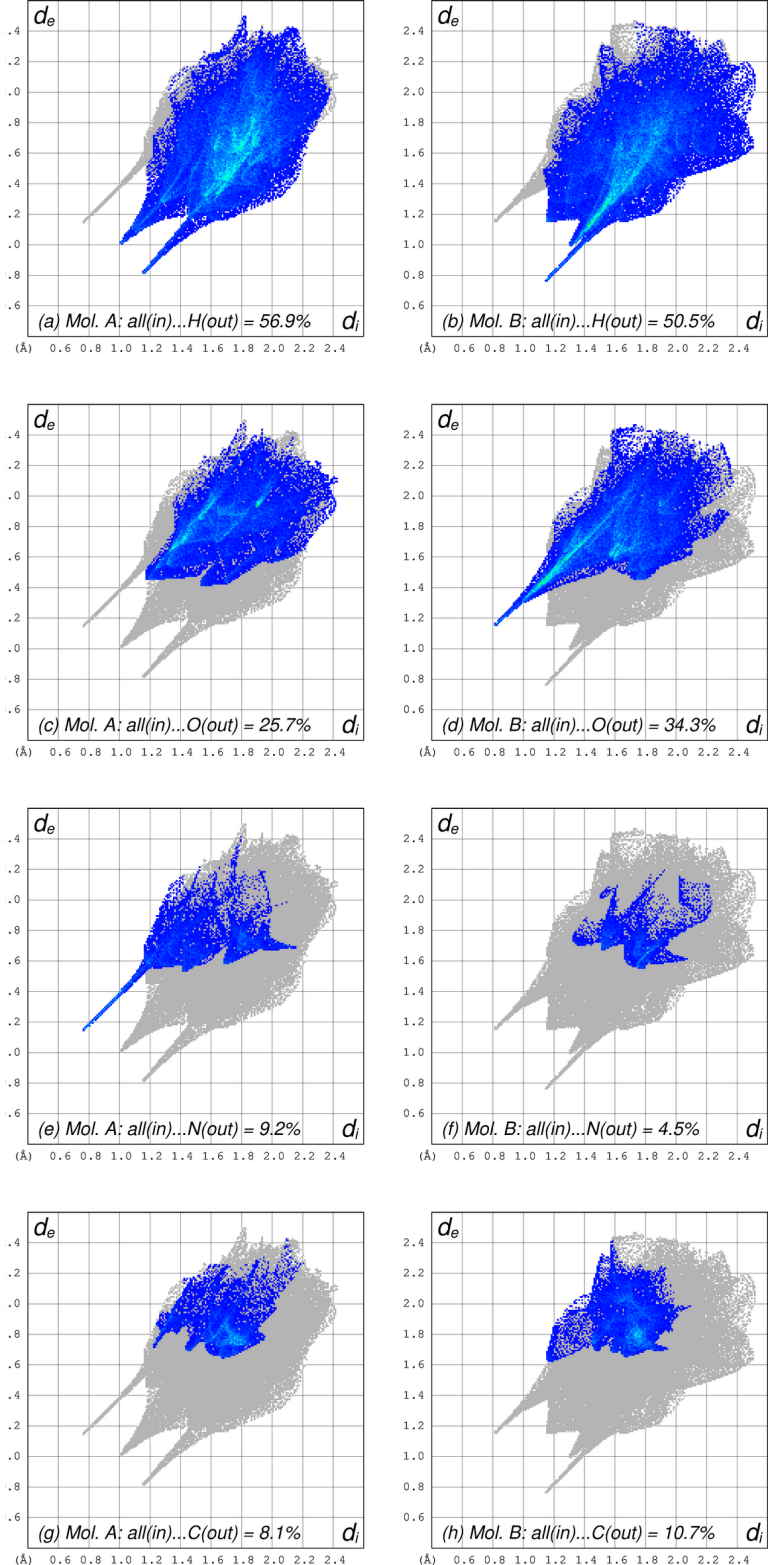
Hirshfeld surface (HS) fingerprint plots calculated independently for mol­ecules *A* and *B*. Panels (*a*) and (*b*) compare contacts between the whole of mol­ecule *A* within its own Hirshfeld surface and hydrogen atoms outside the HS, and *vice versa*. Panels (*c*) and (*d*) show analogous contacts to oxygen atoms, (*e*) and (*f*) show the corresponding plots to nitro­gen, while (*g*) and (*h*) show contacts to carbon.

In **I**, there are only two strong inter­molecular hydrogen bonds: N2*A*—H2*A*⋯N1*B* [*d_D_*_⋯*A*_ = 2.9063 (15) Å] and N2*B*—H2*B*⋯O4*A*^i^ [*d_D_*_⋯*A*_ = 2.9630 (15) Å; symmetry operation: (i) −*x*, −*y* + 1, −*z* + 1], Table 2 ▸. The former connects the two mol­ecules within the chosen asymmetric unit, while the latter generates a centrosymmetric tetra­mer (*i.e.*, a pair of pairs), as shown in Fig. 3 ▸. The integrity of this tetra­mer is augmented by a pair of π–π stacking inter­actions that superimpose the pyrazole ring of mol­ecule *A* with the benzene ring of *B* (plus the equivalent inter­action – symmetry operation i, above), *Cg*⋯*Cg* = 3.524 (1) Å. These tetra­mers stack into columns that propagate parallel to the *a*-axis. In addition, there are a number of weaker hydrogen-bond-type inter­actions of the C—H⋯O form that connect these columns in both the *b-* and *c*-axis directions. The different inter­molecular contacts experienced by mol­ecules *A* and *B* are readily apparent in Hirshfeld surface fingerprint plots (*CrystalExplorer21*, Spackman *et al.*, 2021 ▸). These are shown in Fig. 4 ▸ for mol­ecules *A* and *B* calculated individually, but presented side-by-side for ease of comparison. While it is clear from Fig. 4 ▸*a*,*b* that most inter­molecular contacts involve hydrogen atoms (56.9% and 50.5% for *A* and *B*, respectively), the distributions are different. For *A*, there are no short contacts to oxygen atoms on adjacent mol­ecules (Fig. 4 ▸*c*), whereas for *B* there are (note the sharp blue spike in Fig. 4 ▸*d*). The situation is reversed for contacts to nitro­gen on adjacent mol­ecules (Fig. 4 ▸*e*,*f*). This, of course, is simply a consequence of the different hydrogen-bonding modes of mol­ecules *A* and *B*. The only other types of contact with double-digit percentage coverage are those involving carbon atoms, which are similar, but not identical for *A* and *B* (Fig. 4 ▸*g*,*h*).

## 4. Database survey

A search of the CSD (v5.45 with updates to September 2024; Groom *et al.*, 2016 ▸) of **I** with the nitro and methyl groups removed gave no hits. With the N—H hydrogen also removed, the search returned a single match, 5-methyl-1-[(4-methyl­phen­yl)sulfon­yl]-1*H*-pyrazol-3-yl-4-methyl­benzene sulfonate (CSD refcode XEBLOH; Murtaza *et al.*, 2012 ▸). A search target of 4-nitro­benzene­sulfonate gave 95 hits whereas a search fragment of pyrazol-3-yl sulfonate gave two hits, XEBLOH again, and EBAQUX (Kim *et al.*, 2018 ▸), di-*t*-butyl 3-[(tri­fluoro­methane­sulfon­yl)­oxy]-4,5,7,8-tetra­hydro­pyrazolo­[3,4-d]azepine-1,6-di­carboxyl­ate, which has little else in common with **I**.

## 5. Synthesis and crystallization

**Figure 5:**
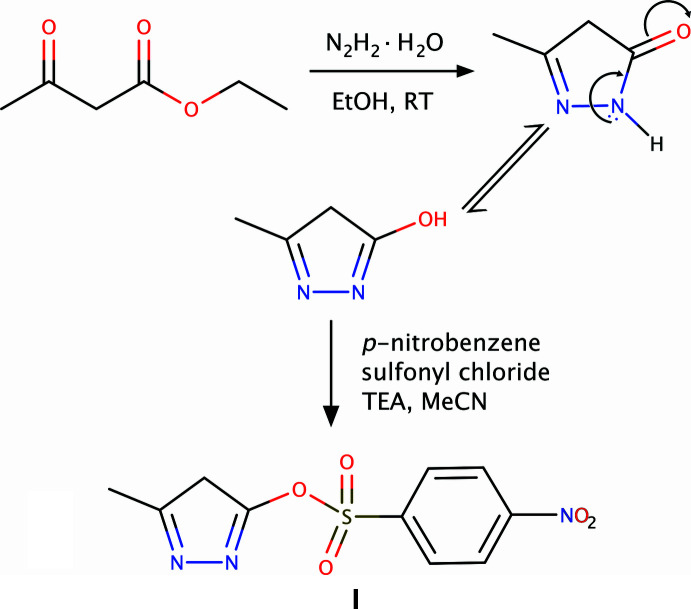
Reaction scheme for the formation of **I**.

An equimolar mixture (0.1 mol) of ethyl aceto­acetate (12.75 ml) and hydrazine hydrate (4.96 ml) in ethanol was stirred for 15–20 min. at room temperature, forming a white precipitate of pyrazolone. The precipitate was then separated by filtration and dried. The pyrazolone (1 g, 10.3 mmol) and 4-nitro­benzene­sulfonyl chloride (2.28 g, 10.3 mmol) were stirred in aceto­nitrile (25 ml) with tri­ethyl­amine for 30 min., turning the reaction mixture yellow–red. Stirring continued for approximately 5 h, with progress monitored by TLC (using hexane and di­chloro­methane as the mobile phase). After acidifying the mixture with 5% HCl, the solvent was evaporated. The product was extracted with ethyl acetate (3 × 15 ml), and the combined organic layers were dried over anhydrous sodium sulfate to yield the crude product, as summarized in Fig. 5 ▸. Recrystallization by slow evaporation from a 1:1 aceto­nitrile–ethyl acetate mixture yielded orange–red crystals after one week.

## 6. Refinement

Crystal data, data collection, and structure refinement details are given in Table 3 ▸. All hydrogen atoms were found in difference-Fourier maps. The N—H hydrogens (*i.e*., H2*A* and H2*B*) were refined freely (*x*, *y*, *z*, *U*_ij_), but carbon-bound hydrogens were included using riding models, with constrained distances set to 0.95 Å (C*sp*^2^H) and 0.98 Å (*R*CH_3_). *U*_iso_(H) parameters were set to values of either 1.2*U*_eq_ or 1.5*U*_eq_ (*R*CH_3_ only) of their attached atom.

## Supplementary Material

Crystal structure: contains datablock(s) I, global. DOI: 10.1107/S205698902401140X/nx2016sup1.cif

Structure factors: contains datablock(s) I. DOI: 10.1107/S205698902401140X/nx2016Isup2.hkl

Supporting information file. DOI: 10.1107/S205698902401140X/nx2016Isup3.cml

CCDC reference: 2404829

Additional supporting information:  crystallographic information; 3D view; checkCIF report

## Figures and Tables

**Table 1:** Conformation-dependant angles and distances (Å, °) in **I**

Torsion angle	Mol­ecule *A*	Mol­ecule *B*
N1—C1—O1—S1	88.97 (12)	83.78 (12)
C1—O1—S1—C5	64.92 (9)	−83.75 (9)
O1—S1—C5—C6	78.91 (10)	95.42 (10)
C7—C8—N3—O5	−0.50 (17)	−6.06 (17)
		
Dihedral angle^*a*^		
*bz*/*nitro*	1.37 (10)	6.78 (4)
*bz*/*pz*	43.10 (4)	37.22 (5)
		
Centroid⋯centroid^*a*^		
*Cg(bz)⋯*Cg*(pz)*	4.505 (1)^*b*^	4.936 (1)^*b*^

Notes: (*a*) Abbreviations: *bz* = benzene; *pz* = pyrazole; *Cg* = centroid. (*b*) These distances do not imply any overlap, they merely show that the rings in *A* are closer than those in *B*.

**Table 2:** Hydrogen bonds and other close contacts (Å, °) in **I**

Hydrogen bonds				
*D*—H⋯*A*	*D*—H	H⋯*A*	*D*⋯*A*	*D*—H⋯*A*
N2*A*—H2*A*⋯N1*B*	0.875 (17)	2.047 (17)	2.9063 (15)	167.0 (15)
N2*B*—H2*B*⋯O4*A*^i^	0.853 (19)	2.121 (19)	2.9630 (15)	169.1 (16)
C2*B*—H2*BA*⋯O2*B*^ii^	0.95	2.63	3.3452 (15)	132.1
C2*B*—H2*BA*⋯O4*B*^iii^	0.95	2.66	3.5201 (15)	151.4
C4*B*—H4*BC*⋯O5*B*^iii^	0.98	2.60	3.5814 (17)	176.8
C6*B*—H6*B*⋯O3*B*^iv^	0.95	2.48	3.3910 (15)	160.1
C7*B*—H7*B*⋯O2*B*^v^	0.95	2.39	3.1622 (15)	137.6
C10*B*—H10*B*⋯O5*B*^v^	0.95	2.54	3.3418 (16)	142.0
				
π–π stacks				
*Ring 1⋯ring 2*		*Distance*	*Dihedral*	
*Cg(pzA)⋯*Cg*(bzB)*		3.524 (1)	5.41 (4)	

Abbreviations: *Cg* = centroid; *bz* = benzene; *pz* = pyrazole. Symmetry codes: (i) −*x*, −*y* + 1, −*z* + 1; (ii) −*x* + 2, −*y*, −*z* + 2; (iii) *x* + 1, *y* − 1, *z*; (iv) −*x* + 1, −*y*, −*z* + 2; (v) *x* − 1, *y*, *z*; (vi) *x* + 1, *y*, *z*.

**Table 3:** Experimental details

Crystal data	
Chemical formula	C_10_H_9_N_3_O_5_S
*M*_r_	283.26
Crystal system, space group	Triclinic, *P* 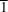
Temperature (K)	90
*a*, *b*, *c* (Å)	7.0823 (3), 11.7865 (6), 15.8999 (8)
α, β, γ (°)	68.340 (1), 81.516 (2), 76.435 (2)
*V* (Å^3^)	1196.39 (10)
*Z*	4
Radiation type	Mo *K*α
μ (mm^−1^)	0.29
Crystal size (mm)	0.28 × 0.21 × 0.14

Data collection	
Diffractometer	Bruker D8 Venture dual source
Absorption correction	Multi-scan (*SADABS*; Krause *et al.*, 2015 ▸)
*T*_min_, *T*_max_	0.930, 0.971
No. of measured, independent and observed [*I* > 2σ(*I*)] reflections	43837, 5472, 4902
*R*_int_	0.039
(sin θ/λ)_max_ (Å^−1^)	0.650

Refinement	
*R*[*F*^2^ > 2σ(*F*^2^)], *wR*(*F*^2^), *S*	0.027, 0.067, 1.04
No. of reflections	5472
No. of parameters	353
H-atom treatment	H atoms treated by a mixture of independent and constrained refinement
Δρ_max_, Δρ_min_ (e Å^−3^)	0.31, −0.42

Computer programs: *APEX5* (Bruker, 2023 ▸), *SHELXT* (Sheldrick, 2015*a* ▸), *SHELXL2019/3* (Sheldrick, 2015*b* ▸), *XP* in *SHELXTL* (Sheldrick, 2008 ▸), *SHELX* (Sheldrick, 2008 ▸) and *publCIF* (Westrip, 2010 ▸).

## supplementary crystallographic information

### 5-Methyl-1H-pyrazol-3-yl 4-nitrobenzenesulfonate Crystal data

**Table:**

C_10_H_9_N_3_O_5_S	*Z* = 4
*M**_r_* = 283.26	*F*(000) = 584
Triclinic, *P*1	*D*_x_ = 1.573 Mg m^−^^3^
*a* = 7.0823 (3) Å	Mo *K*α radiation, λ = 0.71073 Å
*b* = 11.7865 (6) Å	Cell parameters from 9613 reflections
*c* = 15.8999 (8) Å	θ = 2.7–27.5°
α = 68.340 (1)°	µ = 0.29 mm^−^^1^
β = 81.516 (2)°	*T* = 90 K
γ = 76.435 (2)°	Solvent-rounded block, pale orange-brown
*V* = 1196.39 (10) Å^3^	0.28 × 0.21 × 0.14 mm

### 5-Methyl-1H-pyrazol-3-yl 4-nitrobenzenesulfonate Data collection

**Table:**

Bruker D8 Venture dual source diffractometer	5472 independent reflections
Radiation source: microsource	4902 reflections with *I* > 2σ(*I*)
Detector resolution: 7.41 pixels mm^-1^	*R*_int_ = 0.039
φ and ω scans	θ_max_ = 27.5°, θ_min_ = 1.9°
Absorption correction: multi-scan (*SADABS*; Krause *et al*., 2015)	*h* = −8→9
*T*_min_ = 0.930, *T*_max_ = 0.971	*k* = −15→15
43837 measured reflections	*l* = −20→20

### 5-Methyl-1H-pyrazol-3-yl 4-nitrobenzenesulfonate Refinement

**Table:**

Refinement on *F*^2^	Primary atom site location: structure-invariant direct methods
Least-squares matrix: full	Secondary atom site location: difference Fourier map
*R*[*F*^2^ > 2σ(*F*^2^)] = 0.027	Hydrogen site location: mixed
*wR*(*F*^2^) = 0.067	H atoms treated by a mixture of independent and constrained refinement
*S* = 1.04	*w* = 1/[σ^2^(*F*_o_^2^) + (0.0199*P*)^2^ + 0.6984*P*] where *P* = (*F*_o_^2^ + 2*F*_c_^2^)/3
5472 reflections	(Δ/σ)_max_ = 0.001
353 parameters	Δρ_max_ = 0.31 e Å^−^^3^
0 restraints	Δρ_min_ = −0.42 e Å^−^^3^

### 5-Methyl-1H-pyrazol-3-yl 4-nitrobenzenesulfonate Special details

**Table:**

Experimental. The crystal was mounted using polyisobutene oil on the tip of a fine glass fibre, which was fastened in a copper mounting pin with electrical solder. It was placed directly into the cold gas stream of a liquid-nitrogen based cryostat (Hope, 1994; Parkin & Hope, 1998). Diffraction data were collected with the crystal at 90K, which is standard practice in this laboratory for the majority of flash-cooled crystals.
Geometry. All esds (except the esd in the dihedral angle between two l.s. planes) are estimated using the full covariance matrix. The cell esds are taken into account individually in the estimation of esds in distances, angles and torsion angles; correlations between esds in cell parameters are only used when they are defined by crystal symmetry. An approximate (isotropic) treatment of cell esds is used for estimating esds involving l.s. planes.
Refinement. Refinement progress was checked using *PLATON* (Spek, 2020) and by an *R*-tensor (Parkin, 2000). The final model was further checked with the IUCr utility *checkCIF*.

### 5-Methyl-1H-pyrazol-3-yl 4-nitrobenzenesulfonate Fractional atomic coordinates and isotropic or equivalent isotropic displacement parameters (Å^2^)

**Table:**

	*x*	*y*	*z*	*U*_iso_*/*U*_eq_	
S1A	0.65614 (4)	0.71414 (3)	0.53685 (2)	0.01651 (7)	
O1A	0.57056 (13)	0.67119 (8)	0.64020 (6)	0.01783 (18)	
O2A	0.75936 (13)	0.60887 (9)	0.51477 (6)	0.0230 (2)	
O3A	0.75121 (13)	0.81135 (9)	0.53055 (6)	0.0229 (2)	
O4A	−0.13447 (15)	0.85898 (10)	0.31425 (7)	0.0315 (2)	
O5A	−0.16207 (15)	1.02917 (10)	0.34137 (7)	0.0310 (2)	
N1A	0.55538 (15)	0.45947 (10)	0.70016 (7)	0.0172 (2)	
N2A	0.40705 (15)	0.39661 (10)	0.72300 (7)	0.0170 (2)	
H2A	0.434 (2)	0.3155 (16)	0.7480 (11)	0.025 (4)*	
N3A	−0.07568 (16)	0.92479 (11)	0.34531 (7)	0.0230 (2)	
C1A	0.46349 (18)	0.57635 (11)	0.66818 (8)	0.0153 (2)	
C2A	0.26185 (18)	0.59134 (12)	0.66978 (8)	0.0182 (2)	
H2AA	0.168467	0.666642	0.650595	0.022*	
C3A	0.23007 (18)	0.47108 (12)	0.70577 (8)	0.0178 (2)	
C4A	0.0480 (2)	0.42099 (14)	0.7226 (1)	0.0269 (3)	
H4AA	0.010163	0.422405	0.665312	0.040*	
H4AB	−0.056285	0.472441	0.748079	0.040*	
H4AC	0.070410	0.335177	0.765466	0.040*	
C5A	0.44319 (18)	0.77770 (11)	0.47720 (8)	0.0162 (2)	
C6A	0.34479 (19)	0.89710 (12)	0.47076 (9)	0.0202 (3)	
H6A	0.393638	0.944183	0.496873	0.024*	
C7A	0.17492 (19)	0.94661 (12)	0.42591 (9)	0.0213 (3)	
H7A	0.106102	1.028563	0.419575	0.026*	
C8A	0.10808 (18)	0.87335 (12)	0.39054 (8)	0.0186 (2)	
C9A	0.20361 (19)	0.75461 (12)	0.39659 (9)	0.0208 (3)	
H9A	0.153263	0.707371	0.371163	0.025*	
C10A	0.37520 (19)	0.70585 (12)	0.44076 (8)	0.0189 (2)	
H10A	0.444972	0.624470	0.445926	0.023*	
S1B	0.76662 (4)	0.14007 (3)	0.96788 (2)	0.01521 (7)	
O1B	0.82898 (12)	0.13255 (8)	0.86847 (6)	0.01669 (18)	
O2B	0.92921 (13)	0.17288 (9)	0.98930 (7)	0.0225 (2)	
O3B	0.69677 (13)	0.03131 (8)	1.02475 (6)	0.01977 (19)	
O4B	0.15018 (15)	0.68014 (9)	0.87210 (8)	0.0308 (2)	
O5B	−0.05168 (13)	0.55827 (9)	0.89274 (7)	0.0251 (2)	
N1B	0.55988 (15)	0.13086 (9)	0.79934 (7)	0.0161 (2)	
N2B	0.50809 (16)	0.04466 (10)	0.77422 (7)	0.0166 (2)	
H2B	0.400 (3)	0.0622 (16)	0.7499 (12)	0.033 (5)*	
N3B	0.11314 (16)	0.57702 (10)	0.89035 (7)	0.0197 (2)	
C1B	0.72563 (17)	0.07052 (11)	0.83722 (8)	0.0144 (2)	
C2B	0.78320 (18)	−0.05058 (11)	0.83709 (8)	0.0171 (2)	
H2BA	0.896863	−0.109716	0.860015	0.020*	
C3B	0.63631 (19)	−0.06460 (11)	0.79581 (8)	0.0177 (2)	
C4B	0.6056 (2)	−0.17205 (13)	0.77508 (10)	0.0278 (3)	
H4BA	0.621648	−0.153437	0.709356	0.042*	
H4BB	0.473823	−0.187007	0.797278	0.042*	
H4BC	0.701040	−0.246413	0.804956	0.042*	
C5B	0.57217 (17)	0.26894 (11)	0.94752 (8)	0.0143 (2)	
C6B	0.38283 (18)	0.24866 (11)	0.95975 (8)	0.0162 (2)	
H6B	0.358137	0.166606	0.979940	0.019*	
C7B	0.23122 (18)	0.35076 (11)	0.94185 (8)	0.0170 (2)	
H7B	0.099937	0.340364	0.949966	0.020*	
C8B	0.27494 (18)	0.46842 (11)	0.91184 (8)	0.0161 (2)	
C9B	0.46244 (18)	0.48948 (11)	0.89999 (8)	0.0173 (2)	
H9B	0.486532	0.571672	0.879863	0.021*	
C10B	0.61422 (18)	0.38741 (11)	0.91830 (8)	0.0168 (2)	
H10B	0.745178	0.398187	0.910992	0.020*	

### 5-Methyl-1H-pyrazol-3-yl 4-nitrobenzenesulfonate Atomic displacement parameters (Å^2^)

**Table:**

	*U*^11^	*U*^22^	*U*^33^	*U*^12^	*U*^13^	*U*^23^
S1A	0.01586 (14)	0.01781 (15)	0.01583 (15)	−0.00515 (11)	−0.00157 (11)	−0.00453 (11)
O1A	0.0224 (4)	0.0181 (4)	0.0148 (4)	−0.0083 (3)	−0.0019 (3)	−0.0050 (3)
O2A	0.0208 (5)	0.0244 (5)	0.0230 (5)	−0.0009 (4)	−0.0012 (4)	−0.0094 (4)
O3A	0.0215 (5)	0.0239 (5)	0.0241 (5)	−0.0108 (4)	−0.0031 (4)	−0.0048 (4)
O4A	0.0260 (5)	0.0421 (6)	0.0315 (6)	−0.0058 (5)	−0.0115 (4)	−0.0157 (5)
O5A	0.0240 (5)	0.0300 (5)	0.0335 (6)	0.0023 (4)	−0.0068 (4)	−0.0075 (5)
N1A	0.0177 (5)	0.0177 (5)	0.0162 (5)	−0.0041 (4)	−0.0016 (4)	−0.0054 (4)
N2A	0.0192 (5)	0.0135 (5)	0.0177 (5)	−0.0039 (4)	−0.0018 (4)	−0.0041 (4)
N3A	0.0180 (5)	0.0302 (6)	0.0180 (5)	−0.0045 (5)	−0.0015 (4)	−0.0051 (5)
C1A	0.0193 (6)	0.0151 (6)	0.0125 (5)	−0.0054 (5)	−0.0017 (4)	−0.0046 (4)
C2A	0.0180 (6)	0.0168 (6)	0.0177 (6)	−0.0009 (5)	−0.0034 (5)	−0.0042 (5)
C3A	0.0177 (6)	0.0201 (6)	0.0157 (6)	−0.0042 (5)	−0.0022 (5)	−0.0057 (5)
C4A	0.0228 (7)	0.0292 (7)	0.0297 (7)	−0.0109 (6)	−0.0018 (6)	−0.0080 (6)
C5A	0.0171 (6)	0.0178 (6)	0.0130 (5)	−0.0058 (5)	−0.0003 (4)	−0.0033 (5)
C6A	0.0208 (6)	0.0185 (6)	0.0234 (6)	−0.0071 (5)	−0.0025 (5)	−0.0072 (5)
C7A	0.0202 (6)	0.0176 (6)	0.0244 (7)	−0.0034 (5)	−0.0015 (5)	−0.0054 (5)
C8A	0.0155 (6)	0.0247 (6)	0.0139 (6)	−0.0052 (5)	−0.0016 (4)	−0.0037 (5)
C9A	0.0233 (6)	0.0249 (7)	0.0175 (6)	−0.0067 (5)	−0.0034 (5)	−0.0093 (5)
C10A	0.0223 (6)	0.0182 (6)	0.0171 (6)	−0.0036 (5)	−0.0022 (5)	−0.0072 (5)
S1B	0.01556 (14)	0.01389 (14)	0.01669 (15)	−0.00051 (11)	−0.00472 (11)	−0.00597 (11)
O1B	0.0154 (4)	0.0182 (4)	0.0190 (4)	−0.0045 (3)	−0.0003 (3)	−0.0090 (3)
O2B	0.0192 (4)	0.0218 (5)	0.0308 (5)	−0.0003 (4)	−0.0104 (4)	−0.0131 (4)
O3B	0.0245 (5)	0.0140 (4)	0.0180 (4)	−0.0009 (3)	−0.0030 (4)	−0.0035 (3)
O4B	0.0298 (5)	0.0151 (5)	0.0473 (6)	0.0016 (4)	−0.0057 (5)	−0.0130 (4)
O5B	0.0169 (4)	0.0256 (5)	0.0314 (5)	0.0016 (4)	−0.0046 (4)	−0.0106 (4)
N1B	0.0174 (5)	0.0139 (5)	0.0170 (5)	−0.0029 (4)	−0.0024 (4)	−0.0053 (4)
N2B	0.0181 (5)	0.0151 (5)	0.0172 (5)	−0.0030 (4)	−0.0049 (4)	−0.0050 (4)
N3B	0.0199 (5)	0.0171 (5)	0.0216 (5)	0.0006 (4)	−0.0022 (4)	−0.0085 (4)
C1B	0.0155 (5)	0.0148 (6)	0.0131 (5)	−0.0036 (4)	−0.0007 (4)	−0.0048 (4)
C2B	0.0189 (6)	0.0143 (6)	0.0164 (6)	0.0001 (5)	−0.0036 (5)	−0.0046 (5)
C3B	0.0230 (6)	0.0136 (6)	0.0158 (6)	−0.0024 (5)	−0.0034 (5)	−0.0042 (5)
C4B	0.0356 (8)	0.0180 (6)	0.0334 (8)	−0.0036 (6)	−0.0115 (6)	−0.0104 (6)
C5B	0.0158 (6)	0.0142 (5)	0.0133 (5)	−0.0009 (4)	−0.0024 (4)	−0.0058 (4)
C6B	0.0192 (6)	0.0142 (6)	0.0155 (6)	−0.0049 (5)	−0.0002 (5)	−0.0048 (5)
C7B	0.0146 (6)	0.0188 (6)	0.0184 (6)	−0.0040 (5)	0.0001 (5)	−0.0075 (5)
C8B	0.0179 (6)	0.0151 (6)	0.0148 (6)	0.0006 (5)	−0.0018 (4)	−0.0066 (5)
C9B	0.0219 (6)	0.0139 (6)	0.0170 (6)	−0.0050 (5)	−0.0014 (5)	−0.0055 (5)
C10B	0.0167 (6)	0.0173 (6)	0.0179 (6)	−0.0047 (5)	−0.0015 (5)	−0.0070 (5)

### 5-Methyl-1H-pyrazol-3-yl 4-nitrobenzenesulfonate Geometric parameters (Å, º)

**Table:**

S1A—O2A	1.4204 (10)	S1B—O2B	1.4190 (9)
S1A—O3A	1.4260 (9)	S1B—O3B	1.4197 (9)
S1A—O1A	1.6001 (9)	S1B—O1B	1.6063 (9)
S1A—C5A	1.7615 (13)	S1B—C5B	1.7595 (12)
O1A—C1A	1.3977 (14)	O1B—C1B	1.3941 (14)
O4A—N3A	1.2304 (15)	O4B—N3B	1.2235 (14)
O5A—N3A	1.2220 (15)	O5B—N3B	1.2306 (14)
N1A—C1A	1.3190 (16)	N1B—C1B	1.3220 (16)
N1A—N2A	1.3559 (14)	N1B—N2B	1.3578 (14)
N2A—C3A	1.3506 (16)	N2B—C3B	1.3483 (16)
N2A—H2A	0.875 (17)	N2B—H2B	0.853 (19)
N3A—C8A	1.4706 (16)	N3B—C8B	1.4741 (15)
C1A—C2A	1.3948 (17)	C1B—C2B	1.3902 (16)
C2A—C3A	1.3785 (17)	C2B—C3B	1.3793 (18)
C2A—H2AA	0.9500	C2B—H2BA	0.9500
C3A—C4A	1.4905 (18)	C3B—C4B	1.4896 (18)
C4A—H4AA	0.9800	C4B—H4BA	0.9800
C4A—H4AB	0.9800	C4B—H4BB	0.9800
C4A—H4AC	0.9800	C4B—H4BC	0.9800
C5A—C10A	1.3858 (17)	C5B—C10B	1.3891 (17)
C5A—C6A	1.3898 (17)	C5B—C6B	1.3901 (17)
C6A—C7A	1.3831 (18)	C6B—C7B	1.3833 (17)
C6A—H6A	0.9500	C6B—H6B	0.9500
C7A—C8A	1.3838 (18)	C7B—C8B	1.3842 (17)
C7A—H7A	0.9500	C7B—H7B	0.9500
C8A—C9A	1.3789 (18)	C8B—C9B	1.3809 (17)
C9A—C10A	1.3880 (18)	C9B—C10B	1.3838 (17)
C9A—H9A	0.9500	C9B—H9B	0.9500
C10A—H10A	0.9500	C10B—H10B	0.9500
			
O2A—S1A—O3A	120.96 (6)	O2B—S1B—O3B	121.47 (6)
O2A—S1A—O1A	110.09 (5)	O2B—S1B—O1B	103.47 (5)
O3A—S1A—O1A	102.91 (5)	O3B—S1B—O1B	109.58 (5)
O2A—S1A—C5A	109.10 (6)	O2B—S1B—C5B	108.75 (6)
O3A—S1A—C5A	109.78 (6)	O3B—S1B—C5B	109.18 (6)
O1A—S1A—C5A	102.30 (5)	O1B—S1B—C5B	102.69 (5)
C1A—O1A—S1A	117.15 (7)	C1B—O1B—S1B	117.88 (7)
C1A—N1A—N2A	102.28 (10)	C1B—N1B—N2B	102.55 (10)
C3A—N2A—N1A	113.71 (10)	C3B—N2B—N1B	113.21 (10)
C3A—N2A—H2A	127.6 (11)	C3B—N2B—H2B	127.3 (12)
N1A—N2A—H2A	118.7 (11)	N1B—N2B—H2B	119.4 (12)
O5A—N3A—O4A	123.75 (12)	O4B—N3B—O5B	123.91 (11)
O5A—N3A—C8A	118.62 (11)	O4B—N3B—C8B	118.19 (11)
O4A—N3A—C8A	117.62 (11)	O5B—N3B—C8B	117.9 (1)
N1A—C1A—C2A	114.26 (11)	N1B—C1B—C2B	114.10 (11)
N1A—C1A—O1A	119.11 (11)	N1B—C1B—O1B	119.27 (10)
C2A—C1A—O1A	126.58 (11)	C2B—C1B—O1B	126.55 (11)
C3A—C2A—C1A	103.64 (11)	C3B—C2B—C1B	103.64 (11)
C3A—C2A—H2AA	128.2	C3B—C2B—H2BA	128.2
C1A—C2A—H2AA	128.2	C1B—C2B—H2BA	128.2
N2A—C3A—C2A	106.11 (11)	N2B—C3B—C2B	106.49 (11)
N2A—C3A—C4A	122.50 (12)	N2B—C3B—C4B	121.76 (12)
C2A—C3A—C4A	131.36 (12)	C2B—C3B—C4B	131.75 (12)
C3A—C4A—H4AA	109.5	C3B—C4B—H4BA	109.5
C3A—C4A—H4AB	109.5	C3B—C4B—H4BB	109.5
H4AA—C4A—H4AB	109.5	H4BA—C4B—H4BB	109.5
C3A—C4A—H4AC	109.5	C3B—C4B—H4BC	109.5
H4AA—C4A—H4AC	109.5	H4BA—C4B—H4BC	109.5
H4AB—C4A—H4AC	109.5	H4BB—C4B—H4BC	109.5
C10A—C5A—C6A	121.96 (12)	C10B—C5B—C6B	122.51 (11)
C10A—C5A—S1A	119.15 (10)	C10B—C5B—S1B	118.58 (9)
C6A—C5A—S1A	118.86 (10)	C6B—C5B—S1B	118.90 (9)
C7A—C6A—C5A	119.25 (12)	C7B—C6B—C5B	118.43 (11)
C7A—C6A—H6A	120.4	C7B—C6B—H6B	120.8
C5A—C6A—H6A	120.4	C5B—C6B—H6B	120.8
C6A—C7A—C8A	118.10 (12)	C6B—C7B—C8B	118.56 (11)
C6A—C7A—H7A	121.0	C6B—C7B—H7B	120.7
C8A—C7A—H7A	121.0	C8B—C7B—H7B	120.7
C9A—C8A—C7A	123.37 (12)	C9B—C8B—C7B	123.43 (11)
C9A—C8A—N3A	118.64 (12)	C9B—C8B—N3B	118.23 (11)
C7A—C8A—N3A	117.99 (12)	C7B—C8B—N3B	118.33 (11)
C8A—C9A—C10A	118.33 (12)	C8B—C9B—C10B	118.07 (11)
C8A—C9A—H9A	120.8	C8B—C9B—H9B	121.0
C10A—C9A—H9A	120.8	C10B—C9B—H9B	121.0
C5A—C10A—C9A	118.99 (12)	C9B—C10B—C5B	118.99 (11)
C5A—C10A—H10A	120.5	C9B—C10B—H10B	120.5
C9A—C10A—H10A	120.5	C5B—C10B—H10B	120.5
			
O2A—S1A—O1A—C1A	−50.95 (10)	O2B—S1B—O1B—C1B	163.12 (8)
O3A—S1A—O1A—C1A	178.82 (9)	O3B—S1B—O1B—C1B	32.2 (1)
C5A—S1A—O1A—C1A	64.92 (9)	C5B—S1B—O1B—C1B	−83.75 (9)
C1A—N1A—N2A—C3A	0.16 (13)	C1B—N1B—N2B—C3B	0.10 (13)
N2A—N1A—C1A—C2A	0.21 (14)	N2B—N1B—C1B—C2B	0.34 (14)
N2A—N1A—C1A—O1A	177.66 (10)	N2B—N1B—C1B—O1B	177.21 (10)
S1A—O1A—C1A—N1A	88.97 (12)	S1B—O1B—C1B—N1B	83.78 (12)
S1A—O1A—C1A—C2A	−93.93 (13)	S1B—O1B—C1B—C2B	−99.77 (13)
N1A—C1A—C2A—C3A	−0.48 (15)	N1B—C1B—C2B—C3B	−0.64 (14)
O1A—C1A—C2A—C3A	−177.70 (11)	O1B—C1B—C2B—C3B	−177.24 (11)
N1A—N2A—C3A—C2A	−0.46 (14)	N1B—N2B—C3B—C2B	−0.50 (14)
N1A—N2A—C3A—C4A	177.73 (11)	N1B—N2B—C3B—C4B	179.33 (12)
C1A—C2A—C3A—N2A	0.53 (13)	C1B—C2B—C3B—N2B	0.64 (13)
C1A—C2A—C3A—C4A	−177.43 (13)	C1B—C2B—C3B—C4B	−179.16 (14)
O2A—S1A—C5A—C10A	17.63 (12)	O2B—S1B—C5B—C10B	25.67 (11)
O3A—S1A—C5A—C10A	152.3 (1)	O3B—S1B—C5B—C10B	160.25 (9)
O1A—S1A—C5A—C10A	−98.95 (10)	O1B—S1B—C5B—C10B	−83.51 (10)
O2A—S1A—C5A—C6A	−164.5 (1)	O2B—S1B—C5B—C6B	−155.4 (1)
O3A—S1A—C5A—C6A	−29.83 (12)	O3B—S1B—C5B—C6B	−20.82 (11)
O1A—S1A—C5A—C6A	78.91 (10)	O1B—S1B—C5B—C6B	95.42 (10)
C10A—C5A—C6A—C7A	−0.67 (19)	C10B—C5B—C6B—C7B	0.14 (18)
S1A—C5A—C6A—C7A	−178.47 (10)	S1B—C5B—C6B—C7B	−178.75 (9)
C5A—C6A—C7A—C8A	1.16 (19)	C5B—C6B—C7B—C8B	0.55 (18)
C6A—C7A—C8A—C9A	−1.0 (2)	C6B—C7B—C8B—C9B	−0.98 (19)
C6A—C7A—C8A—N3A	178.18 (11)	C6B—C7B—C8B—N3B	178.28 (11)
O5A—N3A—C8A—C9A	178.73 (12)	O4B—N3B—C8B—C9B	−7.47 (17)
O4A—N3A—C8A—C9A	−0.58 (17)	O5B—N3B—C8B—C9B	173.24 (11)
O5A—N3A—C8A—C7A	−0.50 (17)	O4B—N3B—C8B—C7B	173.24 (11)
O4A—N3A—C8A—C7A	−179.80 (12)	O5B—N3B—C8B—C7B	−6.06 (17)
C7A—C8A—C9A—C10A	0.3 (2)	C7B—C8B—C9B—C10B	0.67 (19)
N3A—C8A—C9A—C10A	−178.90 (11)	N3B—C8B—C9B—C10B	−178.59 (11)
C6A—C5A—C10A—C9A	−0.06 (19)	C8B—C9B—C10B—C5B	0.05 (18)
S1A—C5A—C10A—C9A	177.74 (10)	C6B—C5B—C10B—C9B	−0.45 (18)
C8A—C9A—C10A—C5A	0.25 (19)	S1B—C5B—C10B—C9B	178.44 (9)

### 5-Methyl-1H-pyrazol-3-yl 4-nitrobenzenesulfonate Hydrogen-bond geometry (Å, º)

**Table:**

*D*—H···*A*	*D*—H	H···*A*	*D*···*A*	*D*—H···*A*
N2*A*—H2*A*···N1*B*	0.875 (17)	2.047 (17)	2.9063 (15)	167.0 (15)
N2*B*—H2*B*···O4*A*^i^	0.853 (19)	2.121 (19)	2.9630 (15)	169.1 (16)
C2*B*—H2*BA*···O2*B*^ii^	0.95	2.63	3.3452 (15)	132
C2*B*—H2*BA*···O4*B*^iii^	0.95	2.66	3.5201 (15)	151
C4*B*—H4*BC*···O5*B*^iii^	0.98	2.60	3.5814 (17)	177
C6*B*—H6*B*···O3*B*^iv^	0.95	2.48	3.3910 (15)	160
C7*B*—H7*B*···O2*B*^v^	0.95	2.39	3.1622 (15)	138
C10*B*—H10*B*···O5*B*^vi^	0.95	2.54	3.3418 (16)	142

Symmetry codes: (i) −*x*, −*y*+1, −*z*+1; (ii) −*x*+2, −*y*, −*z*+2; (iii) *x*+1, *y*−1, *z*; (iv) −*x*+1, −*y*, −*z*+2; (v) *x*−1, *y*, *z*; (vi) *x*+1, *y*, *z*.
